# A deep learning method for predicting knee osteoarthritis radiographic progression from MRI

**DOI:** 10.1186/s13075-021-02634-4

**Published:** 2021-10-18

**Authors:** Jean-Baptiste Schiratti, Rémy Dubois, Paul Herent, David Cahané, Jocelyn Dachary, Thomas Clozel, Gilles Wainrib, Florence Keime-Guibert, Agnes Lalande, Maria Pueyo, Romain Guillier, Christine Gabarroca, Philippe Moingeon

**Affiliations:** 1 Owkin, 12 Rue Martel, 75010 Paris, France; 2 Servier, Research and Development, 50 rue Carnot, 92284 Suresnes Cedex, France

## Abstract

**Background:**

The identification of patients with knee osteoarthritis (OA) likely to progress rapidly in terms of structure is critical to facilitate the development of disease-modifying drugs.

**Methods:**

Using 9280 knee magnetic resonance (MR) images (3268 patients) from the Osteoarthritis Initiative (OAI) database , we implemented a deep learning method to predict, from MR images and clinical variables including body mass index (BMI), further cartilage degradation measured by joint space narrowing at 12 months.

**Results:**

Using COR IW TSE images, our classification model achieved a ROC AUC score of 65%. On a similar task, trained radiologists obtained a ROC AUC score of 58.7% highlighting the difficulty of the classification task. Additional analyses conducted in parallel to predict pain grade evaluated by the WOMAC pain index achieved a ROC AUC score of 72%. Attention maps provided evidence for distinct specific areas as being relevant in those two predictive models, including the medial joint space for JSN progression and the intra-articular space for pain prediction.

**Conclusions:**

This feasibility study demonstrates the interest of deep learning applied to OA, with a potential to support even trained radiologists in the challenging task of identifying patients with a high-risk of disease progression.

**Supplementary Information:**

The online version contains supplementary material available at 10.1186/s13075-021-02634-4.

## Introduction

Osteoarthritis (OA) is a common disease which constitutes the fourth leading cause of disability worldwide [1]. According to the US National Health Interview Survey, up to 14 million American people are considered to have a symptomatic knee [2], with additional tens of millions affected as well in Europe, South America, Asia, or Middle East [3]. As a consequence of ensuing healthcare expenditures and losses of activity, the economic burden associated with OA is estimated to represent up to 2.5% of Growth National Product in Western countries [4].

The standard of care for OA based on both non-pharmacological and symptomatic pharmacological treatments has only a limited effect on function and pain. Thus, a very high unmet medical need still persists for a disease-modifying osteoarthritis drug (DMOAD) counteracting disease progression for both function and pain and avoiding the requirement for knee surgical replacement. As of today, the development of such DMOADs has been unsuccessful for two reasons. First of all, significant differences are observed among patients in terms of progression of cartilage degradation. Secondly, in the absence of any established patient stratification in the form of endotypes reflecting well-characterized pathophysiological mechanisms, the slow and heterogeneous evolution of the disease makes it difficult to evaluate the effectiveness of a treatment in a broad patient population, within the 1 or 2 year(s) usual timeframe of a clinical study [5].

In this context, a personalized medicine approach is being considered to treat OA, consisting in identifying the most appropriate target populations predicted to benefit from DMOADs [6]. Primary efficacy endpoints required to document DMOAD efficacy include both clinical variables such as requirement for joint replacement as well as structural changes. The diagnosis of knee OA and the evaluation of its severity are currently based on imaging, with radiography remaining the most commonly used modality in clinical practice [7]. Specifically, knee X-rays are used to determine the JSW (joint space width) as a measurement of the distance between tibia and femur considered as an indicator of cartilage thickness. X-rays of the knee performed for an individual patient at various time points allow to define the JSN (joint space narrowing) as a change in JSW over time [8]. Current regulatory guidelines for clinical trials aiming at evaluating candidate DMOADs recommend that JSN should be used as the primary endpoint in those trials [9].

One limitation, however, is that a reliable evaluation of JSN during patient follow-up remains difficult [10]. A clustering method on OAI data during an 8-year follow-up concluded that only 29% of patients displayed a radiographic progression (as defined by JSN), with no further association between progression and pain worsening [11]. In this context, the use of MRI emerges as a better quantitative endpoint recommended for assessing morphological changes in knee cartilage during OA [12]. MRI allows the assessment of meniscal lesions such as root meniscal tears and extrusions known to be associated with OA progression [13, 14]. It also detects other lesions predictive of pain, such as the presence of synovitis and synovial fluid effusion [15] or bone marrow lesions [16].

We thus undertook the present study in support of the development of candidate DMOADs; in order to assess the feasibility of identifying future progressors of knee OA to assess whether knee MR images could predict further cartilage degradation 12 months ahead, we implemented a deep learning method using MR images to build up a predictive model for future progression of knee OA, measured by JSN 12 months after image acquisition. Additional analyses were conducted in parallel to predict pain grade evaluated by the Western Ontario and McMaster Universities Osteoarthritis Index (WOMAC).

## Methods

### Description of the Osteoarthritis Initiative (OAI) database

#### Overview of OAI database

The OAI database is a public multi-center longitudinal database assembled by a consortium led by the National Institutes of Health in the US to help better understand and prevent the progression of knee OA [17]. At baseline, a total of 4796 patients had a bilateral standing knee radiograph (X-ray) and 3D knee MRI. Follow-up visits were done at 12, 24, 36, 48, 72, and 96 months (with 65% of patients enrolled at baseline having a 96 month follow-up visit). The knee MRI sequences include sagittal 3D DESS, coronal 2D IW TSE, and sagittal 2D IW TSE fat-suppressed. A detailed description of MRI sequences from the OAI database can be found in Peterfy et al. [18]. The database further contains clinical information (age, sex, body mass Index [BMI],...), including as well results of pain assessment from WOMAC, a self-administered questionnaire encompassing for each visit up to 24 items divided into 3 subscales (i.e., pain, stiffness and physical function). Assessments from X-rays such as Kellgren & Lawrence (KL) grade [19] and JSW were performed as well in the cohort at several locations in the medial and lateral joint spaces.

#### OAI data analysis

The model was trained on *N* = 9280 knee MR images (2D MRI images of type “COR IW TSE”; detailed information regarding this type of MRI sequence can be found in Peterfy et al. [18]. We used data from 3268 patients (some patients had COR IW TSE knee MRI for both knees whereas others only had COR IW TSE images for a single knee). Those 9280 images were obtained in a sliding window fashion: baseline images were considered to predict month 12 JSN, month 12 images to predict month 24 JSN, and month 24 images to predict month 36 JSN. In light of some data losses (missing JSW measurements, corrupted image data), some images in the OAI database could not be used in the present study. In parallel to those computational analyses, two radiologists (one senior with more than 20 year experience in musculoskeletal imaging and one junior, resident in radiology) were given the task to assess a more restricted set of data corresponding to *N* = 300 baseline knee MRI images.

### Endpoints used in the study

#### Joint space narrowing

OA progression, defined as cartilage degradation over time, was measured by using X-ray images as the minimum JSW in the medial compartment of the knee, with a semi-automated method described in Benichou et al. [20]. This semi-automated measurement was obtained at several time points (baseline, 12 months, 24 months). As proposed by Bruyere et al. [21], a 12-month OA progressor was defined as a patient’s knee exhibiting a JSN at 12 months lower than − 0.5 mm: JSN (*t* + 12 months) = JSW(*t* + 12 months) − JSW(*t*) ≤ − 0.5 mm, where *t* can be baseline, 12 month, or 24 month visits. The threshold of − 0.5 mm for minimum radiographic JSN was identified as clinically relevant in several studies; see Reginster et al. [22] for a review. Since the JSN criteria were evaluated separately for each knee, a patient could be a 12-month OA progressor for a single knee or both. Moreover, a patient could be a progressor for a given knee between baseline and month 12 and then be a non-progressor between month 12 and month 24. Using this JSW variation as a threshold, we proceeded to identify from knee MRIs those patients predicted to lose at least 0.5 mm of knee cartilage.

#### WOMAC pain score

A secondary objective was to study the prediction of pain encoded by the WOMAC score, using contemporary MR images and clinical data (see description of clinical variables in Table 1 in additional materials). Hence, this objective was not to build a model predictive of future evolutions of the disease, but rather to explain the current state of the disease, still exploiting a combination of imaging and clinical information.

#### Evaluation

The performance of models described below was evaluated using a fivefold cross-validation scheme and measured with the following metrics: area under the receiver operating characteristic (ROC AUC score), area under the precision-recall (PR) curve, and F1 score. These metrics are well suited to binary classification tasks which suffer from class imbalance.

### Preprocessing of MRI data

**Fig. 1:**
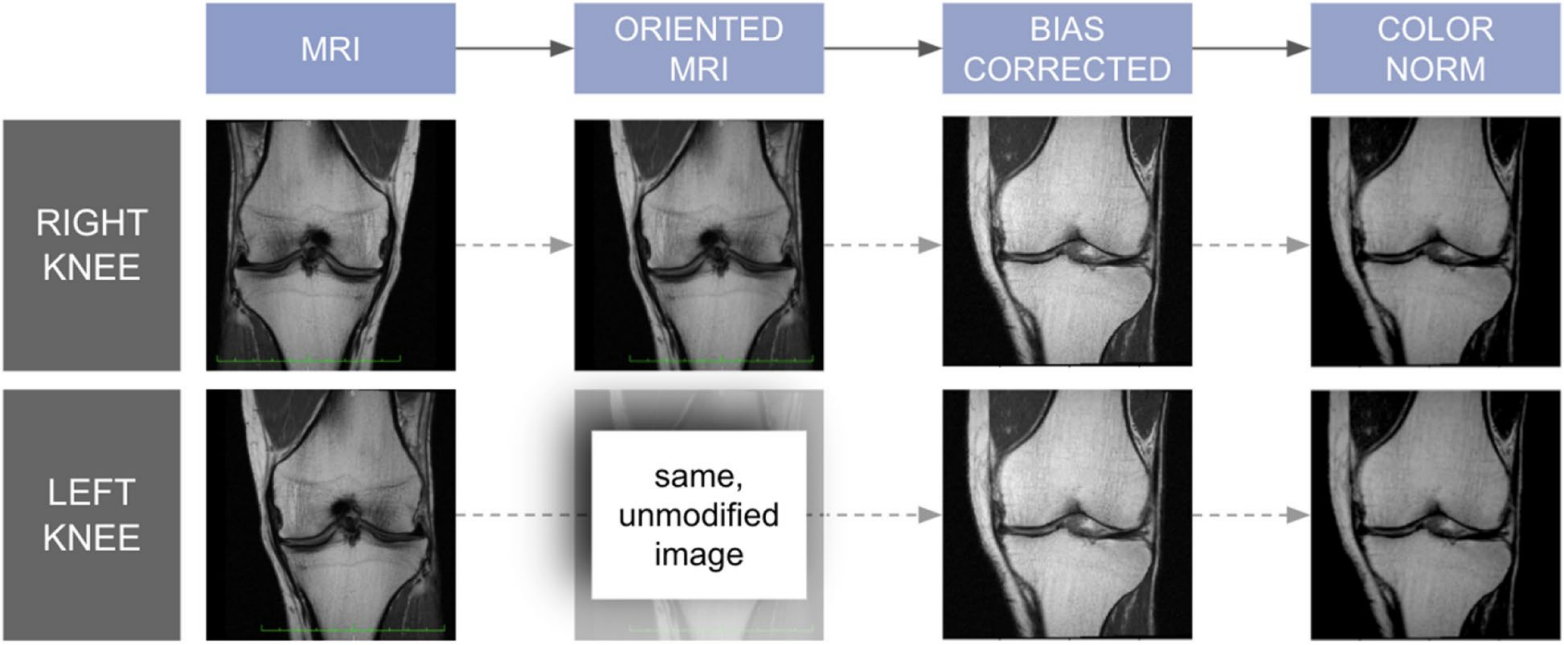
Overview of the image preprocessing pipeline. The raw MR image is first re-oriented so that both left and right knees are similarly oriented. Noteworthy, only the left knee image is flipped, whereas the right is maintained as is, in order to obtain uniform orientations across the dataset. The N4 bias field correction is then applied, followed by a color normalization step

Prior to feeding images into the model, several preprocessing steps have been applied sequentially in order to normalize the dataset, as illustrated in Fig. 1 and summarized below.

#### Image conversion and selection

The OAI database exposes images in the DICOM format. In order to ease image reading and writing operations, each acquisition was converted to the NIFTI format (representing a full, three-dimensional image) by using the dcm2niix software [23].

In order to select images containing most relevant anatomical information, a set of 8 consecutive slices around the middle slice was selected as it provides input slices which contain images of the knee while avoiding the ones with black pixels only.

#### Image orientation

The OAI database contains images of both knees for each patient. Specifically, left knee images are RAS (right, anterior, superior)-oriented, while right knee images are LAS (left, anterior, superior)-oriented. In order to homogenize the dataset, orientations were normalized for all images. To this aim, images of right knees have been “mirrored” along the sagittal-axial plane in order to look similar to images of left knees. This operation was performed using the NiPype python library [24].

#### Bias field correction

MR images can suffer from local magnetic field variations, resulting in artifacts in the reconstructed image. To solve this problem, the N4 bias field correction method [25] was applied to reconstructed images.

#### Color normalization

The final processing step is a color normalization step, which clips out extreme intensity values in the MRI (respectively the 10th and 80th percentiles of the intensity distribution), aiming at erasing bright artifact in the images.

### Model architectures

#### Feature extraction

**Fig. 2:**
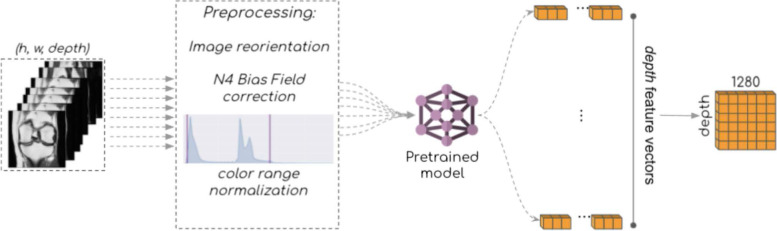
Global overview of the feature extraction step. Converted images undergo several pre-processing steps (reorientation, N4 bias field correction, color normalization) before submitting each slice as input to a pre-trained EfficientNet-B0 network. This neural network will compute 1280 features (or numerical descriptors) for each slice, resulting in depth × 1280 features for an input volume (where depth corresponds to the number of input slices)

In the present study, we used an EfficientNet-B0 network, pre-trained on ImageNet [26] to compute representations of input slices from SAG 3D DESS (or COW/SAG IW TSE) images. Each input slice was converted into a 1280-dimensional feature vector. This approach, most suitable to address problems of very high dimensionality, further allows to speed up model training by delegating the computationally cumbersome task of building meaningful representations from images, prior to feeding those representations into a classification neural network. An overview of the feature extraction process is presented in Fig. 2.

#### Architectures for 2D MRI sequences

Throughout this manuscript, 2D MRI sequences refer to both COR IW TSE and SAG IW TSE images listed in the OAI database. Those images usually contain less slices than 3D sequences such as SAG 3D DESS. Taking this into account, the architecture of the deep learning model used with 2D MRI sequences slightly differs from the ones developed for 3D sequences, as detailed thereafter.

##### Attention sub-model

In light of previous studies related to multiple instance learning, we implemented a gated attention mechanism [27] to compute attention scores (which can be viewed as “importance” scores) for each slice of the input image. Such scores were further used in the second part of the model. Starting from a set of one-dimensional feature vectors for each slice, a 1-dimensional convolution was applied (hence leading to one 2-dimensional matrix per image), followed by a gated recurrent unit (GRU) layer. Such an architecture reduces the 2D matrix to a 1D vector (with one scalar score per input slice). This score was then scaled in the [0, 1] interval through the use of a softmax activation function, thus preventing this sub-model from giving full importance to all slices.

##### Classification sub-model

**Fig. 3:**
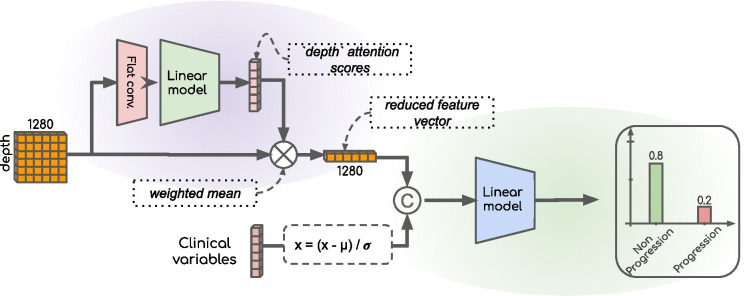
Global overview of the model. The purple-shaded area is a first sub-model aiming to locate regions of interest within input images. The green-shaded area represents the classification sub-model, which aggregates both image and clinical information into progression (or pain score) probabilities

Following calculation of importance scores, a mean weighted by those scores was computed from all feature vectors. Consequently, the model learned to select slices carrying information through the above mentioned attention sub-model. Clinical variables were standardized before being concatenated to this vector, resulting in a 1290-long vector. A description of clinical variables can be found in the additional materials, Table 1. This multimodal 1-dimensional vector was then fed into a multi-layer perceptron (MLP) composed of two hidden layers with a ReLU activation. The MLP was followed by a softmax activation, outputting final class probabilities. In this approach, slices carrying little information (e.g., out-of-knee slices) were given low attention scores, hence participating little (or not at all) to the final logits computed by the second sub-model. A global overview of the model, *from a group of feature vectors* (one per slice of the image) *to the final prediction* (e.g., prediction of progression as an example), is presented in Fig. 3.

### Human benchmark

To further qualify the performance of our predictive model in the identification of 12-month OA progressors, we undertook a comparative study with two expert radiologists, one senior and the other more junior, on the same task. The senior radiologist has specialized in musculoskeletal imaging for more than 20 years whereas the junior radiologist has 2 years of experience. We first selected 300 knee MRI with both SAG 3D DESS, 2D COR IW TSE, and baseline clinical variables (age, gender, BMI, height, weight and minimum JSW in the medial joint space). These 300 knee images were then used to create 150 pairs of knee MRI, each pair being composed of both a 12-month OA progressor and a non-progressor. To account for noise measurement on the minimum medial JSW, the 150 knee MRI of 12-month OA progressors were chosen such that 10 knees were from “almost certain” 12-month OA progressors with JSN (12 months) < − 1.1 mm, 130 satisfied − 1.1 mm ≤ JSN (12 months) < − 0.6 mm, and 10 were “doubtful” progressors with − 0.6 mm ≤ JSN (12 months) < − 0.5 mm. These three classes of progressors reflect the distribution of 12 month JSN in the OAI population. In addition, the − 1.1 mm threshold for “almost certain” 12-month OA progressors was computed using the methodology from Parsons et al. [28]. This threshold takes into account the standard deviation of JSW at baseline and 12 months and further ensures that, with a high probability (≥ 95%), the observed loss of knee cartilage is associated with a degenerative process rather than simply reflecting noise measurement. This methodology mimics the way ROC AUC is computed for a binary classifier [29] as it evaluates the ability of either the radiologists or the classifier to correctly rank two images (picked at random) knowing that one is a positive sample whereas the other one is a negative.

### Model interpretability

In order to get a better understanding of model predictions, the GradCam method [30] was further used to visually pinpoint relevant characteristics within input images, as shown in Fig. 6.

## Results

### Prediction of progression at 12 months

To identify 12-month OA progressors from knee MRI, we developed models to predict JSN (*t* + 12 months) ≤ − 0.5 mm from 2D (SAG IW TSE and COR IW TSE) as well as 3D (SAG DESS) knee MRI sequences. The most promising results were obtained using the classification model depicted in Fig. 3 with slices (8 consecutive slices centered around the middle) from 2D COR IW TSE images and clinical variables (see Table 1 in the additional materials) as input. We thus report below on classification results obtained with 2D COR IW TSE sequences.

**Fig. 4:**
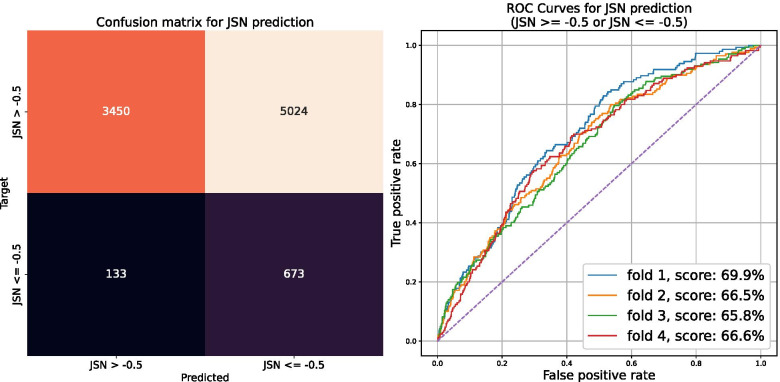
ROC curves and confusion matrix of the binary classification model to identify 12-month OA progressors. The model aims to identify knees for which JSN (*t* + 12 months) ≤ − 0.5 mm. The five curves correspond to the fivefold cross-validation scheme. The dotted diagonal line (purple) illustrates the performance of a random predictor

The performance of our classification model was evaluated using the ROC AUC score, well suited to this task as a metric given the class imbalance: only 9% of the available images are associated with a 12-month OA progression. Using COR IW TSE images, the proposed classification model achieved a ROC AUC score of 65%. This model achieved a precision of 13% and a recall of 84%. The above results are further summarized in a confusion matrix, reported in Fig. 4. Altogether, with SAG 3D DESS images as an input (instead of COR IW TSE), the model achieved a ROC AUC score of 63%.

### Human benchmark

The two radiologists concluded that, for most pairs, their decision was virtually random. Both radiologists found that 2D COR IW TSE volumes were less useful than SAG 3D DESS. The junior radiologist obtained a ROC AUC score of 57.82% whereas the senior radiologist obtained 59.72%. This benchmark with human radiologists highlights the difficulty of identifying 12-month OA progressors using only knee MRI and clinical data at baseline. Nonetheless, these results show the added value of AI in assisting radiologists in a complex image analysis task.

### Prediction of pain severity

We subsequently applied our deep learning approach to the prediction of pain contemporary to image acquisitions. The grading of pain quantified by the WOMAC score was organized into two sets of values, including WOMAC pain score ≥ 2 and WOMAC pain score < 1. The rationale behind this stratification is twofold. On one hand, it reflects some clinical relevance in that pain scores below 2 are often identified as “no pain.” Furthermore, it facilitates a data-driven approach where independent models can be trained and evaluated using only clinical data from different ranges of values. Such models were found to perform better when considering two sets of values with the above mentioned orders of magnitude.

**Fig. 5:**
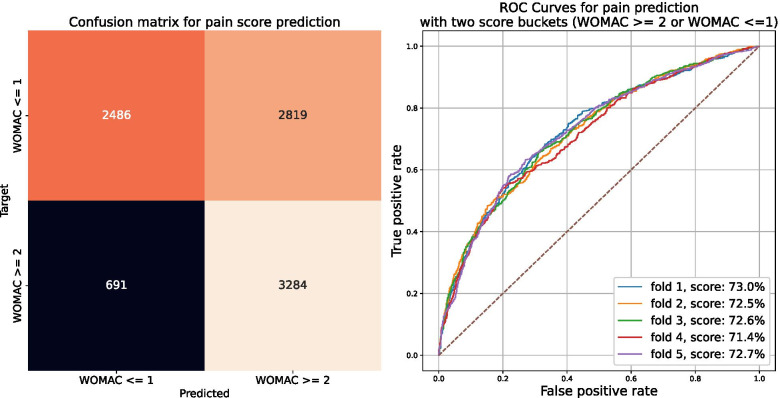
ROC curve and confusion matrix for prediction of pain severity. Graphic representations are shown for the binary task of classifying sets of pain scores across the cross-validation process

Using this approach, our predictive model for pain achieves a mean PR AUC of 66.8% (± 1%), a mean ROC AUC of 72.4% (± 1%) and a mean weighted-F1 score of 65.2% (± 1%). Corresponding ROC curves obtained for each of the five training folds are shown in Fig. 5. For comparison, a random predictor would achieve a mean ROC AUC of 50% and a mean weighted-F1 score of 60%. Globally, the model demonstrated good capabilities to identify high-pain knees (i.e., produce a relatively low number of false negatives), with however a tendency to misclassify non-painful knees (i.e., produce false positives), as can be seen in the confusion matrix represented Fig. 5, left panel.

### Model interpretability

**Fig. 6:**
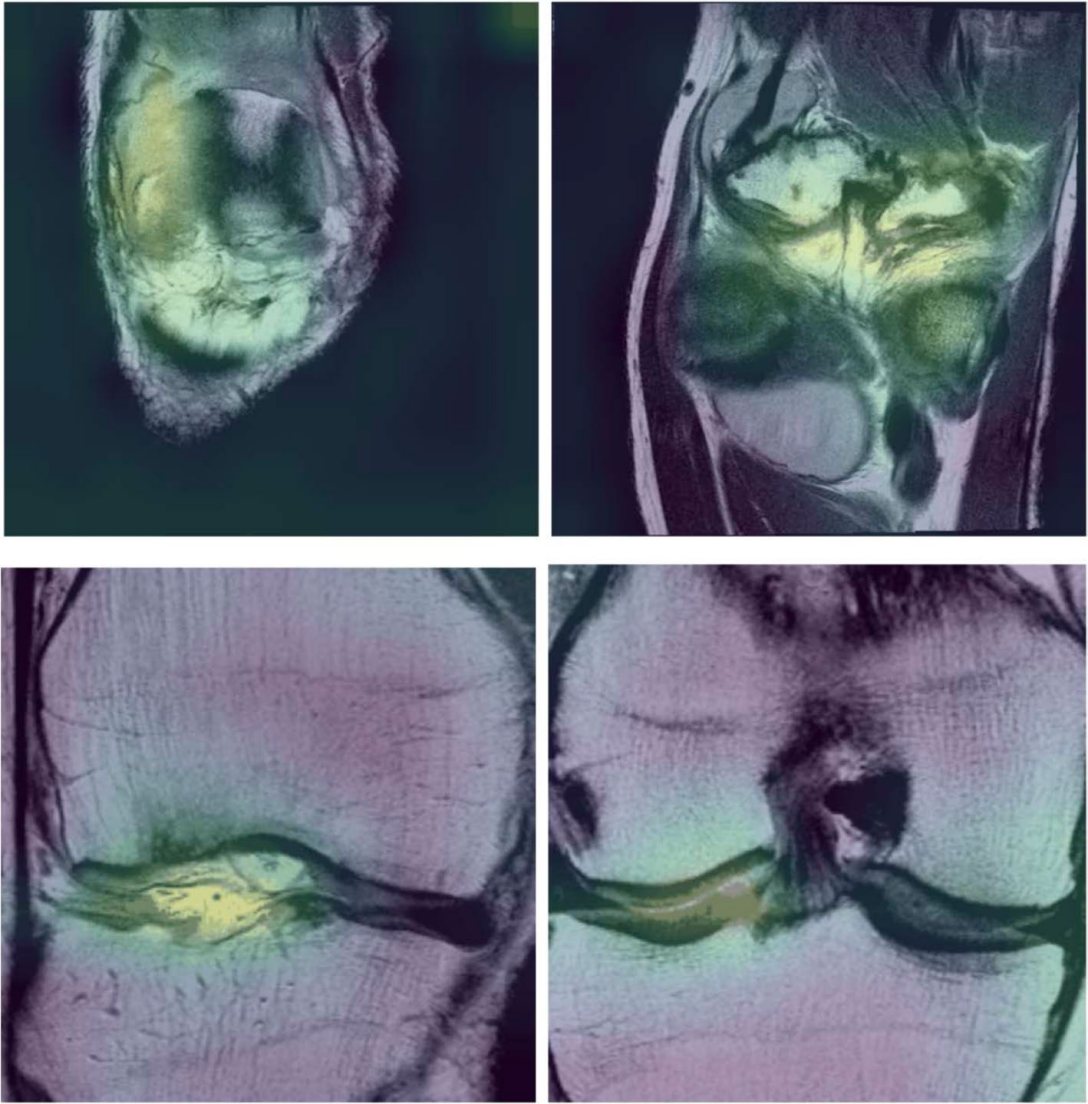
Visual interpretation of relevant zones identified by prediction models. The upper row corresponds to prediction of JSN progression and the bottom row to pain prediction. Yellow areas are the ones considered of high interest by the model: the more intense the yellow, the higher its contribution to a high score for JSN progression prediction (bottom row, coronal view) or severe pain classification (top row, coronal view). All images are obtained from patient 9932578 (right knee)

With the GradCam method (Fig. 6), yellow-colored regions were identified within the joint as the ones contributing with a high probability to the positive class, i.e., progression in the case of JSN progression prediction (Fig. 6, top row), and high WOMAC pain score in the case of pain prediction (Fig. 6, bottom row), respectively. Purple-colored regions did not contribute to high probabilities in the predictions. Interestingly, this analysis emphasized different regions of interest depending on the task. Specifically, JSN progression-related regions are highlighted by the model in the medial joint space. In contrast, for pain prediction, areas of interest are rather located in the intra articular space, where effusion is observed in the case of congestive osteoarthritis (pouches such as suprapatellar pouch and joint spaces).

## Discussion

Predicting disease progression in knee OA is critical to identify patients more likely to benefit from DMOADs and further, to help selecting patients and defining treatment duration in clinical studies evaluating drug candidates [31]. In the present study, we thus developed a weakly supervised deep learning method to build up predictive models for OA progression at 12 months from MR images. Further analyses were also conducted to predict pain grade evaluated by WOMAC from MR images and clinical data at the same visit.

Using COR IW TSE images, our proposed classification model achieved a ROC AUC score of 63%, comparable to the performance of trained radiologists, obtaining a ROC AUC score of 59.72%. To our knowledge, this is the first application of a weak supervised learning method to the prediction of knee osteoarthritis progression from MRI. Although not shown, no improvement on performance was observed on prediction of progression when considering a 24-month follow-up. We also successfully designed a task to identify imaging features associated with pain, leading to a model achieving a ROC AUC score of 72%. This encouraging result is likely explained by the presence of synovial effusion in painful knees, very contrasted in images, and thus easy to detect for a radiologist. Our results are consistent with a previous study relying upon Siamese neural networks to analyze pairs of knees and predict pain with a high AUC (85.3%) [15]. This study confirmed that 86% of correctly predicted painful patients exhibited an effusion-synovitis within areas most associated with pain.

Our study further supports the use of deep learning [32] in musculoskeletal imaging. On 2D radiographs, previous studies have been successfully conducted for bone fracture detection [33], as well as automatic Kellgren and Lawrence Grading for knee OA [34]. Other studies on knee MRI showed strong performance on cartilage segmentation [35], as well as detection or grading of meniscal or anterior cruciate lesions [36]. All these studies relied upon “strong” labeling methods, requiring time-consuming manual image annotations by expert radiologists, in contrast to the deep learning approach reported here.

Deep learning methods are often described as “black-boxes,” referring to the lack of interpretability of their predictions. Interpretability can however be introduced in the form of “heatmaps” generated using a GradCam method [30] to highlight the relevant regions in the knee MRI used by the predictive model. In our study, such attention modeling of OA progression confirmed the importance of internal joint space, consistent with the fact that the joint space narrowing is evaluated in this anatomic compartment. The pain prediction model rather showed heatmaps focused on the intra-articular space, where cartilage, meniscal lesions, and effusion synovitis are observed. In future developments, other interpretability methods based on generative adversarial networks (GANs) could be applied to generate synthetic imaging features reflecting pathophysiologic processes of interest in OA. Whereas GANs modeling the natural history of OA progression observed on knee radiographs have been developed [37], such studies remain to be done on MRI.

Other developments in AI-based image analyses could be considered to improve the predictive models obtained in our feasibility study. For example, whereas we used MRI as inputs for predicting an endpoint determined from knee X-ray imaging, further studies could rather use MRI criteria as endpoints of progression in clinical trials of knee osteoarthritis rather than joint space narrowing > 0.5 mm, which is a criteria difficult to quantify reproducibly. In this regard, we investigated, in a post hoc analysis, the use of different criteria to characterize OA progression. Whereas our initial analyses have been based on “absolute” JSN (12 months) ≤ − 0.5 mm, reflecting that a knee is a “12-month OA progressor” when the minimum JSW is reduced by 0.5 mm in the medial joint space of the knee, we reasoned that this “absolute” criteria may not be suited to knees with advanced OA. We thus considered as an alternative a “relative” criteria defined by: JSN (*t* + 12 months) ≤ − 25% relative to JSW(t), with a threshold value chosen in order to ensure that the dataset has approximately the same class imbalance as with the “absolute” criteria. In this approach, a classification model based on 2D COR IW TSE and validated using a 5-fold cross-validation strategy obtained an average ROC AUC score of 80%, suggesting an interest in considering relative over absolute JSN reduction as an alternative endpoint of OA progression.

## Conclusions

The deep learning approach developed herein is based on “weak” labels for machine learning tasks, i.e., relying on information not explicitly shown in images as targets for predictions, in combination with clinical variables such as BMI (a “multimodal” approach). This data-driven methodology, which intends to predict the future evolution of a disease, is providing information that cannot be directly assessed in the clinical routine of a radiologist. This proof of concept shows the added value of deep learning in clinical practice as it applies to OA, with the promise of a convergence of intelligences between machines and radiologists in the interpretation of radiological images [38, 39]. The future in the field is likely one of a new era of augmented radiology.

## Supplementary Information

**Additional file 1: Table 1.** Listing and description of the clinical variables used in predictive models. **Figure 7.** Average One-vs-One PR-AUC as a function of threshold pairs (T_1_, T_2_)= (threshold1, threshold2). Each tile is colored according to the average of PR-AUC scores (5-fold CV) obtained using the 853 clinical variables at baseline (all the clinical variables available at baseline, except those associated with a self-evaluation of pain). **Figure 8.** Confusion matrix for the three-classes WOMAC pain score. Rows correspond to “true” classes whereas columns correspond to predicted classes. Class 0 versus 1 or 2: Precision of 64%, recall of 90% and F1 of 75%. Class 2 versus 0: Precision of 76%, recall of 20% and F1 of 32%.

## Data Availability

The dataset used in the current study are publicly available from the OAI database (The Osteoarthritis Initiative 2002 [cited 2021 Jan 26]. Available from: https://nda.nih.gov/oai/).

## Abbreviations

DMOAD: Disease-modifying osteoarthritis drugs
GRU: Gated recurrent unit
JSN: Joint space narrowing
JSW: Joint space width
KL grade: Kellgren and Lawrence grade
OA: Osteoarthritis
OAI: The Osteoarthritis Initiative database
WOMAC: Western Ontario and McMaster Universities Arthritis Index
ROC AUC: Area under the receiver operating characteristic
PR/PR-AUC: Precision recall/area under the precision-recall curve
GAN: Generative adversarial networks
MR(I): Magnetic resonance (imaging)
ML: Machine learning
