# Supplementary material for: A deep learning method for predicting knee osteoarthritis radiographic progression from MRI
Source: Arthritis Res Ther. 2021 Oct 18;23:262. doi: 10.1186/s13075-021-02634-4 (PMC8521982; doi:10.1186/s13075-021-02634-4)
Supplement: Supplementary file 1 — Additional file 1: Table 1. Listing and description of the clinical variables used in predictive models. Figure 7. Average One-vs-One PR-AUC as a function of threshold pairs (T1, T2)= (threshold1, threshold2). Each tile is colored according to the average of PR-AUC scores (5-fold CV) obtained using the 853 clinical variables at baseline (all the clinical variables available at baseline, except those associated with a self-evaluation of pain). Figure 8. Confusion matrix for the three-classes WOMAC pain score. Rows correspond to “true” classes whereas columns correspond to predicted classes. Class 0 versus 1 or 2: Precision of 64%, recall of 90% and F1 of 75%. Class 2 versus 0: Precision of 76%, recall of 20% and F1 of 32%. [file 13075_2021_2634_MOESM1_ESM.docx]

ADDITIONAL MATERIALS

# 1. Description of clinical variables

Unless stated otherwise, predictive models use a subset of clinically relevant variables along with MR images.

| **Variables** | **Description** |
| --- | --- |
| *P01HEIGHT* | Patient’s height (cm) |
| *P01WEIGHT* | Patient’s weight (kg) |
| *V00CSPACE* | JSW at baseline (mm) |
| *V0020MPACE* | 20-meter walk pace (m/s) |
| *V00XROSFM* | OARSI grade in the femur medial joint space (0-3) |
| *V00XROSFL* | OARSI grade in the femur lateral joint space (0-3) |
| *V00XROSTM* | OARSI grade in the tibia medial joint space (0-3) |
| *V00XROSTL* | OARSI grade in the tibia lateral joint space (0-3) |
| *V00AGE* | Age at baseline (y) |
| *P02SEX* | Patient’s sex (M/F) |

*Table 1: Listing and description of the clinical variables used in predictive models.*

# 2 Digitizing WOMAC pain score

In order to elect a pair of thresholds which determine three classes of pain scores, the following approach was adopted:

Considering a pair of thresholds $T_{1}, T_{2}$: a linear model was trained on all 853 baseline clinical data to predict the class of pain scores defined by the aforementioned thresholds. Using a k-folds cross-validation scheme, this model was evaluated on the whole population using the ROC AUC score, PR AUC score and F1 score.

Given two distinct threshold values $T_{1}, T_{2}$such that $T_{1}<T_{2}$, one can define three classes of WOMAC pain score. Having $T_{1}$close to $T_{2}$($T_{2}-T_{1}<1$for example) is not particularly interesting since few individuals will fall into the class satisfying to the condition: $T_{1}\leq$WOMAC pain score $<T_{2}.$Therefore, we consider a range of possible threshold values for $T_{1}, T_{2}$ and enforce the condition $T_{2}-T_{1}>2$ to avoid a “degenerate” class 1. [Figure 7](#bookmark=id.3tbugp1) presents the average AUC scores (5-fold cross-validation) obtained for all pairs of threshold values $(T_{1},T_{2})$ satisfying the condition $T_{2}-T_{1}>2$. This figure suggests that the thresholds pair $(T_{1},T_{2})$ which provides the “best” multi-class classification performance is $(T_{1},T_{2}) = (1,8)$. However, other pairs could be used with a similar (or close) classification performance.


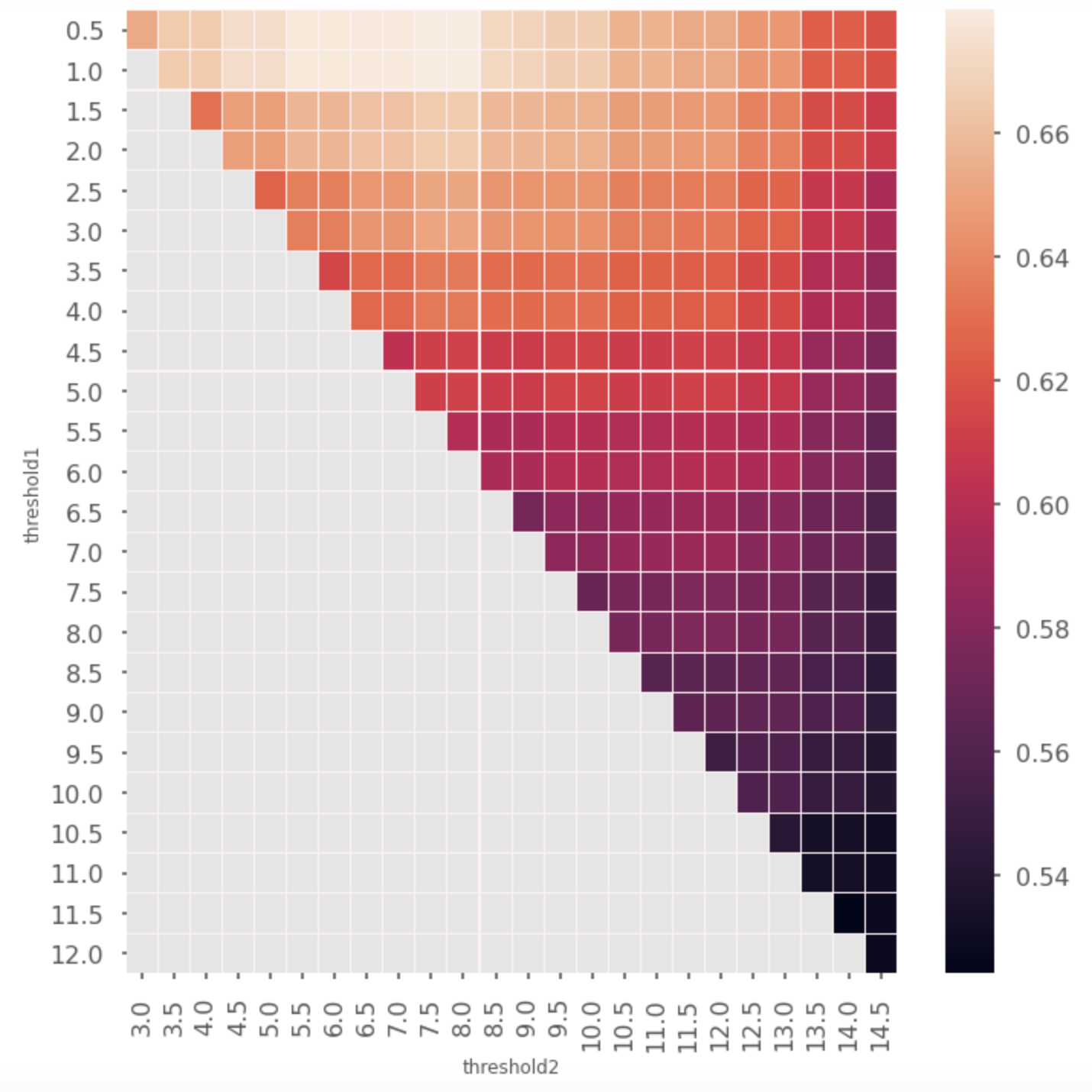


*Figure 7: Average One-vs-One PR-AUC as a function of threshold pairs* $(T_{1},T_{2})$*= (threshold1, threshold2). Each tile is colored according to the average of PR-AUC scores (5-fold CV) obtained using the 853 clinical variables at baseline (all the clinical variables available at baseline, except those associated with a self-evaluation of pain).*

# 3 Supplementary results on prediction of pain severity

## 3.1 With three buckets of score

The approach presented here was also evaluated against the prediction of pain, quantified by the WOMAC score, organized into three buckets of value: WOMAC < 2, 2 ≤ WOMAC < 8 and 8 ≤ WOMAC.

The rationale behind those pain score buckets is two-folds:

1. It follows a clinical reality in which pain scores above 8 are often considered severe, while scores equal to zero or one are often identified as “no pain”.
2. It follows a data driven approach, where independent models were trained and evaluated using only clinical data with different bucket values, as described in the second section of the additional materials. It was found that such models were performing better with bucket values of this order of magnitude.

In this case, the model achieves a mean PR AUC of 65,7% (+/- 1%), along with a mean ROC AUC of 80,4% (+/- 7.2%) and a mean weighted-F1 score of 55.4% (+/- 1%). A confusion matrix illustrating those results on the whole cohort can be found in Figure 8.


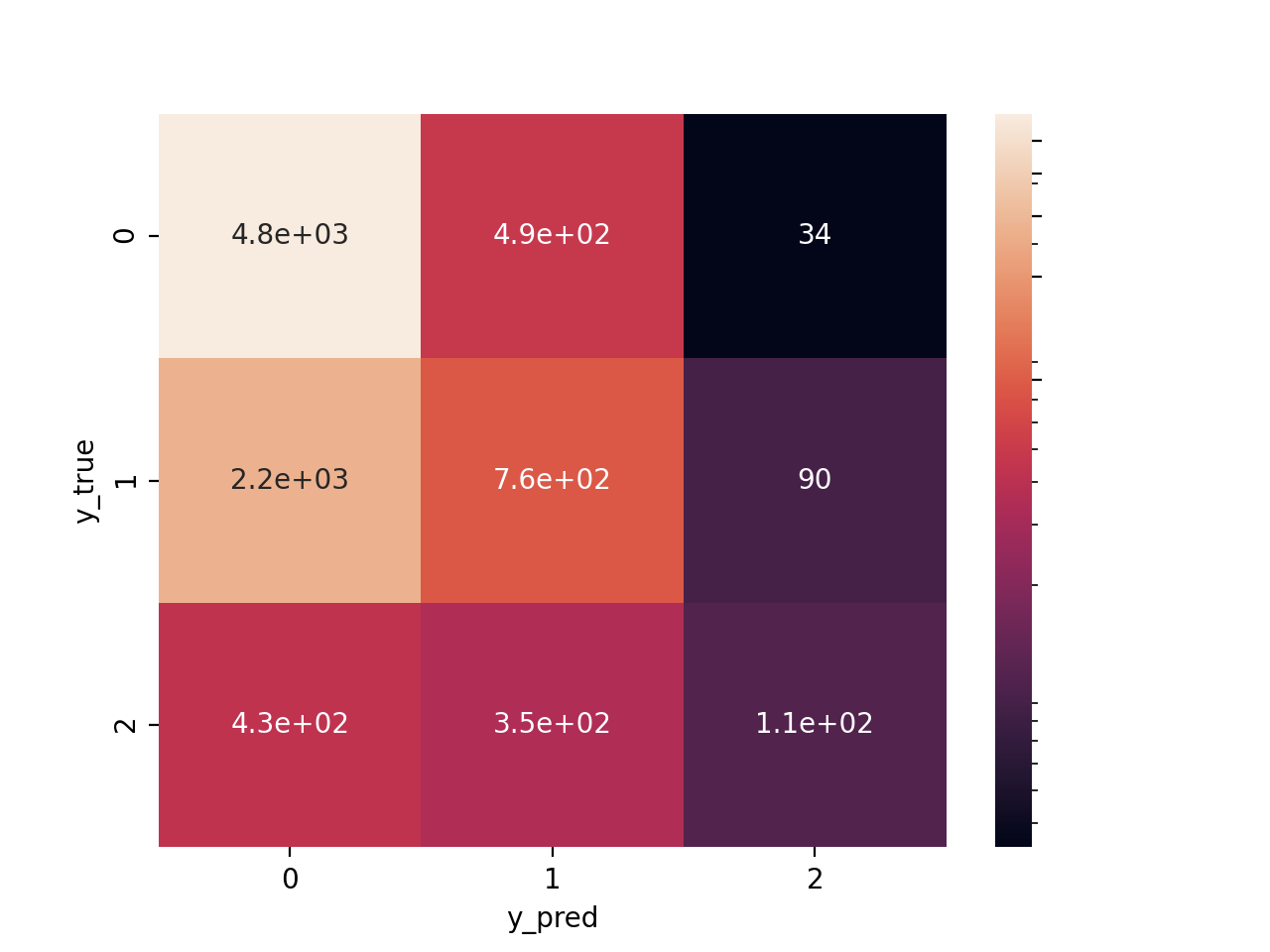


*Figure 8: Confusion matrix for the three-classes WOMAC pain score. Rows correspond to “true” classes whereas columns correspond to predicted classes. Class 0 versus 1 or 2: Precision of 64%, recall of 90% and F1 of 75%. Class 2 versus 0: Precision of 76%, recall of 20% and F1 of 32%.*
